# Structural and Synaptic Organization of the Adult *Reeler* Mouse Somatosensory Neocortex: A Comparative Fine-Scale Electron Microscopic Study of *Reeler* With Wild Type Mice

**DOI:** 10.3389/fnana.2018.00080

**Published:** 2018-10-05

**Authors:** Miriam Prume, Astrid Rollenhagen, Joachim H. R. Lübke

**Affiliations:** ^1^Institute of Neuroscience and Medicine INM-10, Research Centre Jülich GmbH, Jülich, Germany; ^2^Department of Psychiatry, Psychotherapy and Psychosomatics, Medical Faculty, RWTH University Hospital Aachen, Aachen, Germany; ^3^JARA Translational Brain Medicine, Jülich, Germany

**Keywords:** adult *reeler* mouse, somatosensory neocortex, neuronal clusters, synaptic organization, fine-scale electron microscopy

## Abstract

The *reeler* mouse has been widely used to study various aspects of cortico- and synaptogenesis, but also as a model for several neurological and neurodegenerative disorders. In contrast to development, comparably little is known about the neuronal composition and synaptic organization of the adult *reeler* mouse neocortex, in particular at the fine-scale electron microscopic level, which was investigated here and compared with wild type (WT) mice. In this study, the “barrel field” of the adult *reeler* and WT mouse somatosensory neocortex is used as a model system. In *reeler* the characteristic six-layered structure is no longer existent, but replaced by a conglomerate of neurons organized in homologous clusters with maintained morphological identity and heterologous clusters between neurons and/or oligodendrocytes. These clusters are loosely scattered throughout the neocortical mass between the pial surface and the white matter. In contrast to WT, layer 1 (L1), if existent, seems to be diluted into the volume of the neocortical mass with no clear boundary. L1 also contains clusters of migrated or persistent neurons, oligodendro- and astrocytes. As in WT, myelinated and unmyelinated axons were found throughout the neocortical mass, but in *reeler* they were organized in massive fiber bundles with a high fiber packing density. A prominent and massive thalamocortical projection traverses through the neocortical mass, always accompanied by numerous “active” oligodendrocytes whereas in WT no such projections were found and “silent” oligodendrocytes were restricted to the white matter. In the adult *reeler* mouse neocortex, synaptic boutons terminate on somata, dendritic shafts, spines of different types and axon initial segments with no signs of structural distortion and/or degeneration, indicating a “normal” postsynaptic innervation pattern of neurons. In addition, synaptic complexes between boutons and their postsynaptic targets are tightly ensheathed by fine astrocytic processes, as in WT. In conclusion, the neuronal clusters may represent a possible alternative organization principle in adult *reeler* mice “replacing” layer formation. If so, these homologous clusters may represent individual “functional units” where neurons are highly interconnected and may function as the equivalent of neurons integrated in a cortical layer. The structural composition and postsynaptic innervation pattern of neurons by synaptic boutons provide the structural basis for the establishment of a functional although altered cortical network in the adult *reeler* mouse.

## Introduction

The *reeler* mouse is an autosomal recessive mutation and was first described by Falconer (1951), Hamburgh (1963) and later more extensively by Caviness and co-workers (Caviness et al., 1972; Caviness and Sidman, 1973; Pinto-Lord and Caviness, 1979). After the discovery of the extracellular matrix glycoprotein *Reelin*, this mouse became an attractive model to study neuronal migration (Hashimoto-Torii et al., 2008; Schaefer et al., 2008; Britto et al., 2011; Honda et al., 2011; Bosch et al., 2016; Chai et al., 2016) as well as cortico- and synaptogenesis (Rice and Curran, 2001; Hammond et al., 2010; Ventruti et al., 2011; Bosch et al., 2016). *Reeler* mice, lacking functional *Reelin* exhibit severe cytoarchitectonic malformations causally related to the disturbed neuronal migration of neurons into the neocortex (D’Arcangelo et al., 1995; Hirotsune et al., 1995; Ogawa et al., 1995). It was shown by earlier studies that migrating neurons, although maintaining normal apposition to radial glial fibers, were finally unable to bypass these fibers and post-migratory neurons with their leading process when entering the neocortex. Moreover, migrating neurons abnormally adhere much stronger to radial glial fibers during the period of neuronal differentiation and growth in *reeler* (Caviness and Rakic, 1978; Pinto-Lord et al., 1982). More recent studies demonstrated that by binding the two lipoprotein receptors, apolipoprotein E receptor (ApoER2) and very low-density lipoprotein receptor (VLDLR), *Reelin* interacts with the Notch-pathway, thereby regulating radial migration, polarity and branching of newborn excitatory neurons along radial glial fibers (Borrell et al., 1999; Trommsdorff et al., 1999; Hack et al., 2007; Hashimoto-Torii et al., 2008; Chai et al., 2015, 2016). The role of *Reelin* is even more complex. Beside acting as a stop signal causing neurons to detach from radial glial fibers (Frotscher, 1998), the mutation of *Reelin* leads to cerebellar hypoplasia and the malpositioning of neurons (Goffinet et al., 1984; Lambert de Rouvroit and Goffinet, 1998; D’Arcangelo, 2005). Recent studies using highly specific molecular marker-based phenotyping approaches revealed novel features of abnormal “lamination” in the* reeler* mouse. Instead of a rough laminar inversion, as long thought, the* reeler* neocortex is disrupted and distorted in a much more complex way with no layering (Wagener et al., 2010; Guy et al., 2015). Moreover, a mirror-image laminar phenotype and cell-type specific differences between cortical areas were demonstrated (Boyle et al., 2011). In addition, various other anatomical anomalies were described in a number of different CNS areas in *reeler* (reviewed by Katsuyama and Terashima, 2009).

Meanwhile, numerous studies have investigated various aspects of brain development in *reeler*; but comparably little, in particular no comprehensive data, are known about the structural composition and synaptic organization of the adult *reeler* mouse (but see Watanabe et al., 1995; Soriano et al., 1997; Alcántara et al., 1998; Coulin et al., 2001; Cremer et al., 2011; Ventruti et al., 2011; Pielecka-Fortuna et al., 2015). Hence, the aim of the present study was to investigate the structural composition and synaptic organization of the adult *reeler* mouse neocortex using the “barrel field” of the somatosensory area as a model system by means of high-resolution, fine-scale electron microscopy (EM) and to compare the findings with the wild type (WT) somatosensory neocortex.

Here, we demonstrate that in *reeler* neurons, which are no longer arranged in their respective layers, form homologous clusters with other neurons and heterologous clusters with oligodendrocytes throughout the entire neocortical mass, the area between the pial surface and white matter. Second, massive myelinated and unmyelinated axonal fiber bundles are present throughout the neocortical mass, which are not present in WT mice. A prominent thalamocortical projection ascends from the white matter to L1, runs parallel to the pial surface and then loops down to the deep neocortical mass. These bundles are often associated with “active” oligodendrocytes and neuronal clusters, a scenario not found in WT mice. Finally, the composition of synapses and their postsynaptic innervation pattern remain “unaltered” and thus comparable with that shown in the WT mouse neocortex (for example see Rodriguez-Moreno et al., 2018).

## Materials and Methods

All experimental procedures were approved by the Animal Research Committee of the Research Centre Jülich GmbH and complied with the guidelines laid out in the EU directive regarding the protection of animals used for experimental and scientific purposes (2004/23/EC).

### Fixation and Tissue Processing

For the light microscopic and EM experiments, adult (8 month and older) WT (Strain C57/Bl6; *n* = 5) and *reeler* (Strain Orleans^−/−^; *n* = 5) mice were deeply anesthetized with Narkodorm™ (60 mg/kg body weight), and then transcardially perfused through the ascending aorta with 0.1 M phosphate-buffered (PB) saline for 1–2 min at a constant flow rate (8 ml/min) using a rotation pump (SCI 323, Watson-Marlow, Rommerskirchen, Germany). This was followed by an ice-cold fixative containing 4% paraformaldehyde and 0.1 or 0.5% glutaraldehyde for 15 min. Brains were removed from the skull, post-fixed for 1 h in the same but fresh fixative at 4°C and were afterwards extensively washed in PB and stored overnight in PB at 4°C. The next day coronal sections (150 μm in thickness) were cut using a vibratome (VT1000S; Leica Microsystems GmbH, Bensheim, Germany) and collected in PB. After post-fixation for 1 h in sucrose-PB containing 1% osmium tetroxide, sections were washed in PB and dehydrated in an ascending series of ethanol to absolute ethanol starting with 20% ethanol. Afterwards sections were transferred to propylene oxide (twice 2 min each), then into a mixture of propylene oxide and epoxy resin (2:1, 1:1; Durcupan™; ACM, Fluka, Neu-Ulm, Germany) for 1 h, and finally stored overnight in pure resin. The next day they were flat-embedded in fresh Durcupan™ between coated glass slides, coverslipped and polymerized at 60°C for 2 days.

From the embedded *reeler* and WT material, blocks containing the “barrel field” of the somatosensory neocortex were trimmed, semithin sections were cut, toluidine-blue stained and examined light microscopically to identify the region of interest (ROI). Then serial ultrathin sections (~20–80 sections/series; ~55 ± 5 nm in thickness, silver to light gray interference contrast appearance) were cut through the ROI with an ultramicrotome (UltracutS; Leica Microsystems GmbH, Wetzlar, Germany). The sections were collected on Pioloform-coated slot copper grids (Plano, Munich, Germany). After counterstaining with an aqueous 5% uranyl acetate (5–20 min) and lead citrate solution (2–7 min; Reynolds, 1963) grids were examined with a Libra 120 electron microscope (Carl Zeiss GmbH, Germany) equipped with a bottom-mounted ProScan 2K digital camera (Albert Tröndle, Moorenweis, Germany) and the SIS Analysis software (Olympus Soft Imaging Solutions GmbH, Münster, Germany). Digital images were taken at various magnifications using the SIS Multi Images Acquisition or Image SP software (Albert Tröndle, Moorenweis, Germany), and were finally stored in databases.

For documentation, all specimens were examined light microscopically and photographed using a motorized Olympus BX61 microscope equipped with the Olympus CellSense analysis hard- and software (Olympus GmbH, Hamburg, Germany). For publication selected light microscopic- and EM images were further processed using Adobe Photoshop™ and Adobe Illustrator™ software packages.

## Results

### Cytoarchitectural Organization of the Adult *Reeler* Mouse Neocortex

It has been clearly demonstrated by numerous studies that the characteristic six-layered structure of the neocortex including the “barrel field” of the somatosensory area in adult WT mice undergoes severe alterations in its cytoarchitecture in the adult *reeler* mouse neocortex as revealed by Nissl-, cytochrome oxidase staining and layer-specific cDNA probes (Caviness et al., 1976; Welt and Steindler, 1977; Wagener et al., 2010, 2016; Cremer et al., 2011). Despite the lack of layers, a prominent although altered “barrel field” is existent in adult *reeler* as in WT (Wagener et al., 2010, 2016). Within the neocortex, neurons with varying sizes and morphologies are no longer organized in layers but in homologous neuronal clusters (for details see below).

### Cytoarchitectonic Organization of Fiber Tracts in the *Reeler* and WT Neocortex

**Figure 1:**
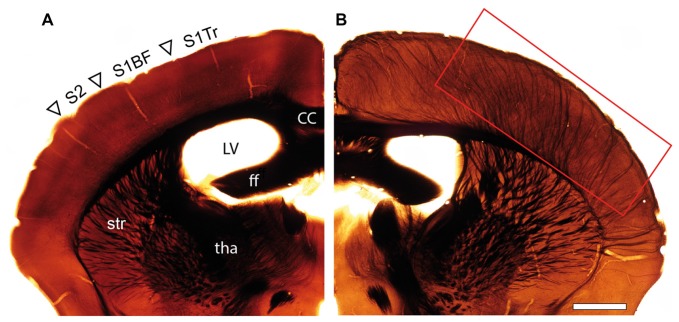
Organization of fiber tracts in the adult wild type (WT) and *reeler* mouse. **(A,B)** Comparison of an adult WT **(A)** and *reeler* **(B)** mouse coronal hemisphere after osmium-treatment and embedding for EM. Note the massive fiber bundles (framed area) in somatosensory and motor areas in the *reeler* mouse neocortex. Abbreviations in **(A)**: cc, corpus callosum; ff, fimbria fornix; LV, lateral ventricle; tha, thalamus; str, striatum; S1Tr, primary somatosensory cortex trunk region; S1BF, primary somatosensory cortex “barrel field”; S2, secondary somatosensory cortex. Scale bar 1 mm.

Osmium-treated EM-embedded material of adult WT (Figure 1A) and *reeler* (Figure 1B) mice, showed severe changes in the organization and orientation of major fiber tracts in *reeler* mice. In particular, the presence of a prominent thalamocortical projection in the mutant brain is completely absent in WT mice (compare Figure 1A with Figure 1B). These massive fiber bundles originate from the somatosensory relay nuclei, the ventral posteromedial nucleus and the posteromedial complex of the thalamus (Rodriguez-Moreno et al., 2018; reviewed by Clascá et al., 2016), and traverse through the corpus striatum and white matter before entering the neocortex (Figure 1B).

### Fiber Tracts in the Adult *Reeler* and WT Mouse Neocortex at the Fine-Scale EM Level

**Figure 2:**
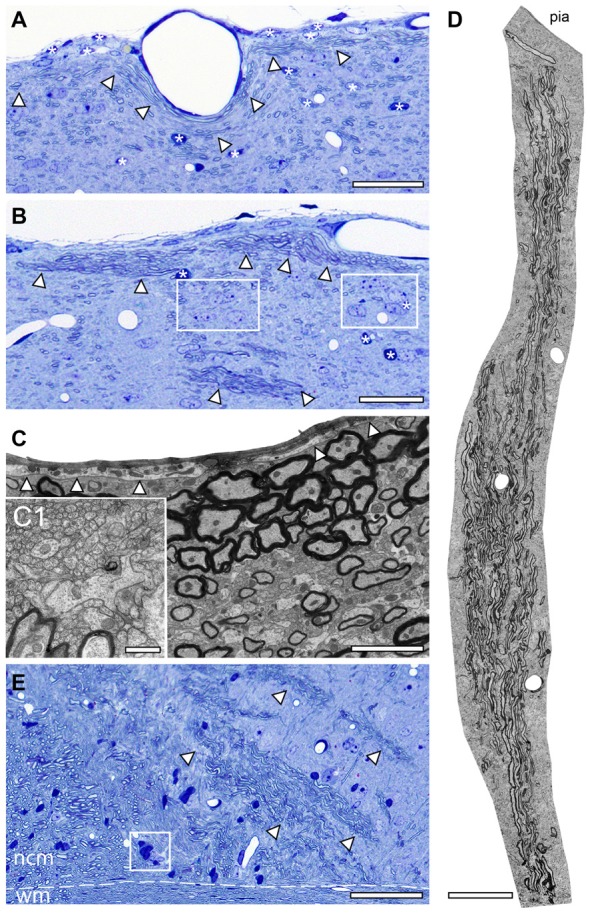
Axonal fiber bundles in the adult *reeler* mouse neocortex. **(A,B)** Toluidine-blue stained semithin sections through the most superficial part directly underneath the pial surface of the adult *reeler* somatosensory neocortex. Note the long-range horizontal myelinated axons (arrowheads) running parallel to the pial surface and the scattered neurons some of which are organized in clusters (framed areas in **B**). Some astro- and oligodendrocytes are marked by asterisks. Scale bar in **(A,B)** 100 μm. **(C)** EM micrograph showing the density and distribution of myelinated and unmyelinated axons located close and running parallel to the pial surface. The basal lamina of the glia limitans is marked by arrowheads. Scale bar 2 μm. **(C1)** Myelinated fibers are always accompanied by bundles of unmyelinated axons shown at higher magnification. Scale bar 0.5 μm. **(D)** EM photomontage of a large axonal fiber bundle from the pial surface (pia) to deep neocortical mass. Note the packing density of the myelinated axons. Scale bar 200 μm. **(E)** Toluidine-blue stained semithin section at the level of the white matter (wm)/neocortical mass (ncm) border (dashed line). Several fiber bundles (marked by arrowheads) arise from the white matter and ascend to different regions within the neocortical mass. Framed area shows a heterologous cluster of neurons and astrocytes. Scale bar 100 μm.

**Figure 3:**
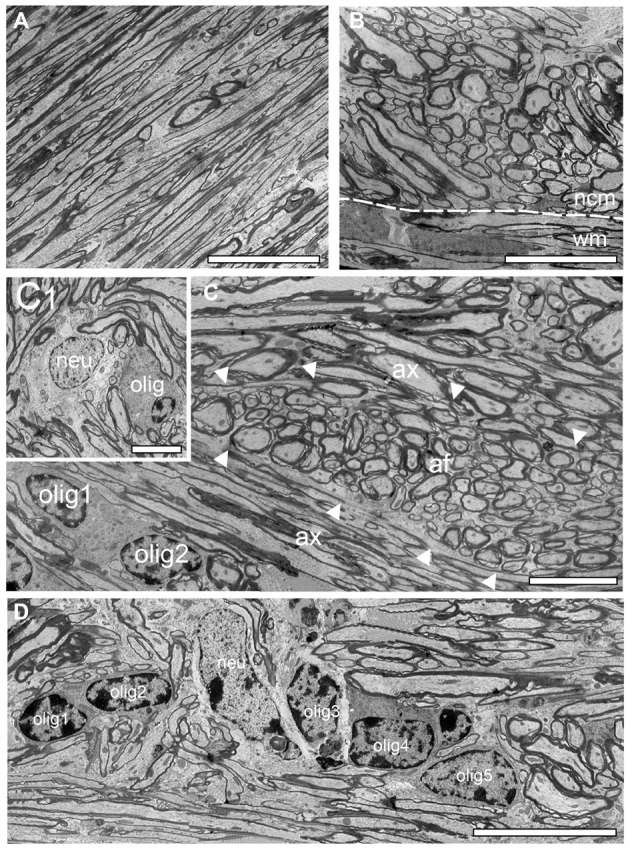
Structural organization of the white matter *reeler*. **(A)** High power EM micrograph of parallel running axonal bundles in the deep white matter. Scale bar 50 μm. **(B)** Transition zone between the white matter (wm) and the neocortical mass (ncm; dashed line) with emerging putative ascending thalamocortical axons traversing the neocortical mass. Note the change in the orientation of the myelinated axons. Scale bar 50 μm. **(C)** Fasciculated axonal fibers (af, marked by arrowheads) between parallel oriented white matter axons (ax) with two oligodendrocytes (olig1, olig 2). Scale bar 25 μm. **(C1)** Within such fascicles white matter neurons (neu) and oligodendrocytes (olig) were sometimes observed. Scale bar 10 μm. **(D)** Five oligodendrocytes (olig 1–5) building a heterologous cluster with a neuron (neu) in deep white matter. Scale bar 20 μm.

As already mentioned above, a striking feature of the investigated adult *reeler* mouse “barrel cortex” was the existence of massive ascending and descending myelinated axonal fiber bundles throughout the neocortical mass (Figures 1B, 2). Thalamic fiber bundles directly passed through the white matter and then ascended through the neocortical mass with different orientations (Figures 2E, 3B). In the most superficial part of the neocortical mass, myelinated axons formed prominent horizontally oriented fiber bundles (projections) running parallel directly underneath the pial surface (Figures 2A–C). Clusters of unmyelinated axons of different shape and size often accompanied these myelinated fibers (Figures 2C,C1). These subpial myelinated and unmyelinated axonal fiber bundles were always characterized by their high packing density within the surrounding neuropil (Figures 2B,C,C1); in contrast to their density and distribution pattern in WT mice. In the most superficial part of the neocortical mass the majority of these axonal bundles completely changed their orientation in *reeler* (Figures 2C,D). Axonal fiber bundles were seen traversing the entire neocortical mass from the deep neocortical mass to the subpial surface (Figure 2D). Such massive ascending and descending axonal fiber bundles were never observed and described in the neocortex of adult WT mice at the semithin and EM level (this study).

**Figure 4:**
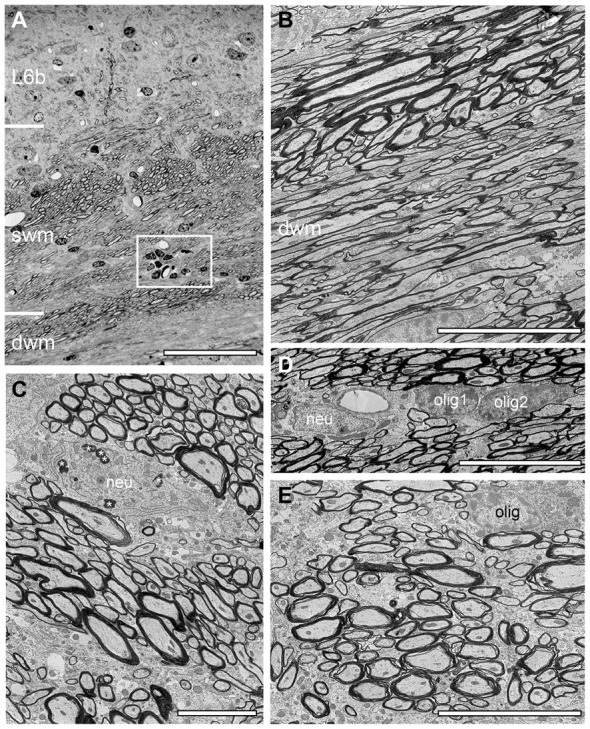
Structural composition of the white matter in WT mouse neocortex. **(A)** Semithin section at the L6/white matter border. Note the abrupt loss of neurons at the L6/white matter border, the different orientation of axonal fiber bundles in the superficial (swm), deep white matter (dwm) and the occurrence of a cluster of oligodendrocytes (framed area). Scale bar 50 μm. **(B)** EM micrograph showing part of the dwm with axonal fibers of different orientation, shape and size. Scale bar 10 μm. **(C)** EM micrograph through the swm showing individual fascicles with the same fiber orientation. Occasionally also neurons (neu) were found within such fascicles, some of which contained lipofuscin granula (asterisks). Scale bar 5 μm. **(D)** Heterogeneous group of two oligodendrocytes (olig1, olig2) and a neuron (neu) in dwm. Scale bar 5 μm. **(E)** High-power EM micrograph of an individual axonal fascicle in the swm with a neighboring oligodendrocyte (olig). Scale bar 5 μm.

Besides the fiber bundles, single or clusters of neurons together with astro- and oligodendrocytes were located in the most superficial part of the neocortex in *reeler* (Figures 2A,B). In contrast, oligodendrocytes and such prominent fiber bundles were never observed in the gray matter of WT mice where myelinated axons seemed to be randomly distributed throughout all layers of the neocortex, but not in such high densities despite in L6B when compared with *reeler* (Figure 4A).

### Structural Composition of the Neocortical/White Matter Border in *Reeler* and WT Mice

As described for adult WT mice the white matter in *reeler* is composed of massive myelinated axonal fiber bundles (compare Figure 3 with Figure 4) that could be followed over long distances separating the entire neocortex from the underlying hippocampus. In some regions at the gray matter/white matter border in both* reeler* and WT mice, a dense accumulation of axonal fibers was seen to emerge from the white matter, but with a completely different orientation than the majority of white matter axons running parallel to the gray matter/white matter border (Figures 3, 4). These fibers then fan out to ascend into the neocortex (Figures 2E, 3B, 4A,B) in both *reeler* and WT mice. In addition, within the white matter periodically axonal fascicles of variable shape, size and orientation were found, intermingled with parallel running axons (Figures 3B,C, 4B,C,E). However, in contrast to *reeler* these massive fasciculated fiber bundles were restricted to the L6B/white matter border and do not invade the other cortical layers in WT in such high packing density (compare Figure 2E with Figure 4A). Such fascicles were often accompanied by oligodendrocytes, although neurons were also infrequently observed in both *reeler* and WT mice (Figures 3C,C1,D, 4C,D). Homologous clusters of several oligodendrocytes were frequent in the deep white matter in *reeler* and WT and were found in close proximity to the white matter axons. At the gray matter/white matter border and in deep white matter, smaller and mainly heterologous clusters of at most 2–5 neurons (distinguishable from oligodendrocytes by their shape and size, the lighter appearance of their nucleus, arrangement of their heterochromatin and cytoplasm) were found intermingled with oligodendrocytes (Figures 3C1,D) in *reeler*. Infrequently white matter neurons were also found in WT mice intermingled with axonal fascicles, but more at the L6B/white matter border (Figures 4A,C).

### Neuronal and Synaptic Organization of the Adult *Reeler* and WT Mouse Neocortex Structural Composition of the Most Superficial Part Close to the Pia Mater

**Figure 5:**
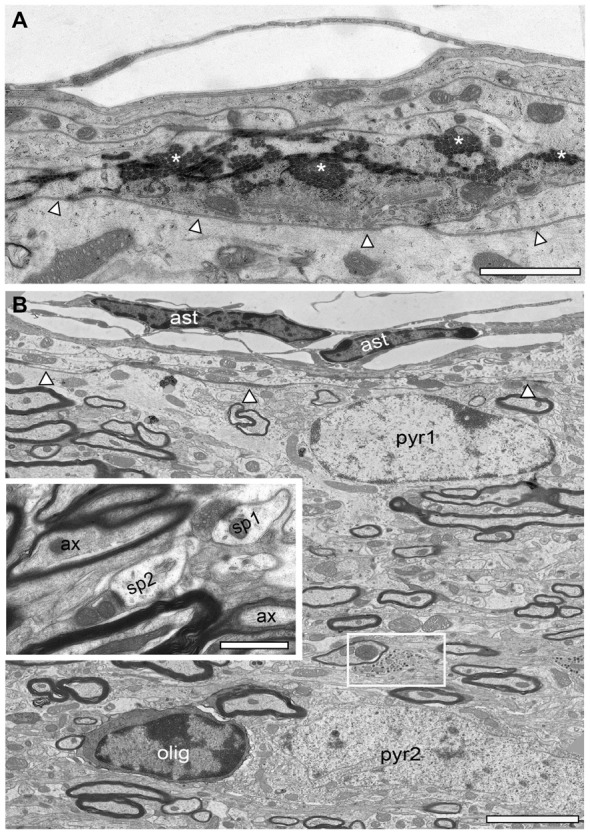
Structural organization of the adult *reeler* mouse neocortex underneath the pial surface. **(A)** Low EM micrograph of the most superficial part underneath the pia mater of the *reeler* mouse neocortical mass. Note the long horizontally oriented bundles of collagen fibers (asterisks). The basal lamina is outlined by arrowheads. Scale bar 1 μm. **(B)** Overview of the structural composition of the most superficial part in the adult *reeler* mouse neocortex. Here, the neuropil contains migrated, putative pyramidal neurons (pyr1, pyr2). Beside neurons, oligodendrocytes were also observed (olig) often associated with neurons and always with axonal bundles running parallel to the pial surface. Note a specialized type of synapses (small framed area) with high numbers of dense core vesicles predominantly found in this area shown at higher magnification in Figure 9B. Abbreviation: ast, astrocytes. The basal lamina is outlined by arrowheads. Inset, between the massive axonal bundles (ax), dendrites, spines (sp1, sp2) and synaptic boutons of various shape and size were regularly found. Scale bar 0.5 μm.

**Figure 6:**
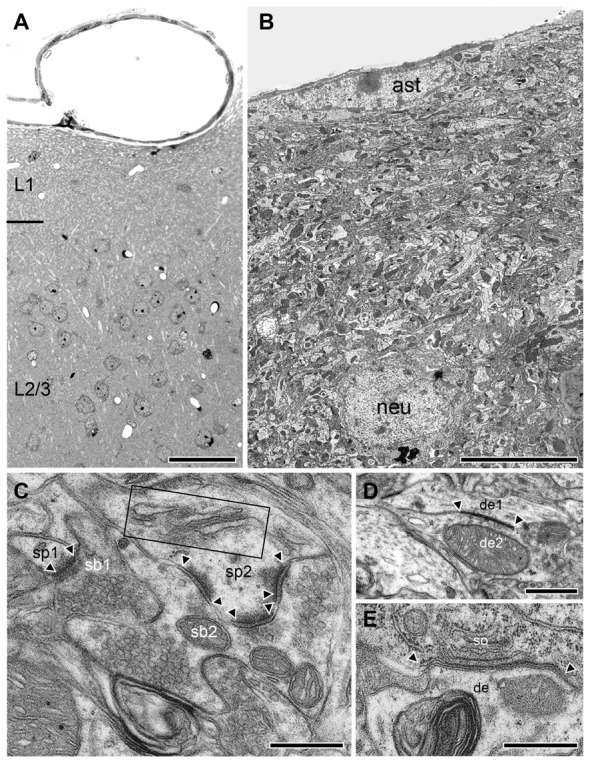
Structural organization of L1 in the adult WT neocortex. **(A)** Semithin section trough the superficial part of the WT neocortex from the pial surface to the superficial part of L2/3. Note the absence of neurons in L1 in this image but their abrupt occurrence in the underlying L2/3. Scale bar 10 μm. **(B)** Low power EM micrograph from the pial surface to deep L1 showing the organization of the neuropil including small caliber axons, dendrites and synapses of different shape and size. Infrequently GABAergic interneurons (neu) were found identifiable by their typical nuclear enfolding. Note also the astrocytes (ast) with its fine processes located directly underneath the pial surface. Scale bar 10 μm. **(C)** High power EM micrograph of two adjacent synaptic boutons (sb1, sb2) terminating on two different spines (sp1, sp2) in L1. The smaller (sp1) had only a single active zone whereas the larger spine (sp2) contained a spine apparatus (framed area) and three active zones (arrowheads). Scale bar 0.25 μm. **(D)** Tight-junction (arrowheads) between two adjacent dendrites (de1, de2). Scale bar 0.5 μm. **(E)** Gap-junction (arrowheads) between the soma (so) and a dendrite (de). Scale bar 0.5 μm.

The pia mater is the delicate innermost layer of the meninges that firmly adheres to the surface of the brain and loosely connects to the arachnoid layer in both *reeler* (Figures 2A,B, 5) and WT (Figure 6). In adult *reeler* and WT mice, the pia mater is one or two cell layers thick and composed of flat connective tissue separated from the underlying neuronal tissue by extracellular matrix, namely loose collagen fibers (Figure 5A). In addition, it is anchored to the brain by a fine dense network of protoplasmic astrocytes in *reeler* (Figure 5B) and WT (Figure 6B) building the glia limitans that joins with the underlying ependym containing ependymal cells, pericytes and sometimes macrophages. These cells often contained lysosomes and peroxisomes as internal organelles. The glia limitans delineates the border to the underlying neocortex (Figure 5B) and was found directly underneath the basal lamina (Figure 5B). In adult *reeler*, a direct contact between neurons and the superficial basal lamina bordering the glia limitans layer was observed (Figures 2A, 5B; see also Derer, 1979) but not seen and described for WT mice (Figures 6A,B).

In adult *reeler*, the most superficial part that may represent L1 is not a cell-sparse zone when compared to adult WT mice (compare Figure 5B with Figures 6A,B) where only various types of GABAergic interneurons are present (Figure 6B; Muralidhar et al., 2013; Yao et al., 2016). L1 is more compact in *reeler* as shown for example in Nissl-staining (Caviness et al., 1976; Welt and Steindler, 1977). Beside astro- and oligodendrocytes (Figures 2A, 5B) L1 contained various neuronal cell types such as migrated putative pyramidal neurons (Figure 5B; for identification of neuronal cell types at the EM level see Peters and Kara, 1985; Peters and Sethares, 1991; Peters et al., 1991) and various types of GABAergic interneurons (Figure 6B). In the superficial part, excitatory neurons often formed homologous clusters of variable size (3–5 neurons; Figure 2B), but also heterologous clusters of oligodendrocytes and neurons were found (Figure 2A). Neurons belonging to individual clusters receive synaptic inputs at their somata, dendritic shafts and spines of unknown origin, one requirement for their integration in a functional cortical network. No such clusters or even single oligodendrocytes were found throughout all cortical layers in adult WT mice where they are restricted exclusively to the white matter (Figures 4A,D). In addition, the superficial neuropil was largely comprised of dendrites and spines of different caliber, shape, size and orientation in both *reeler* and WT mice (compare Figure 5B with Figures 6A,B). Beside dendrites, synaptic boutons were distributed throughout the neuropil; sometimes located within the myelinated und unmyelinated fiber bundles (Figure 5B inset, Figure 9B).

**Figure 7:**
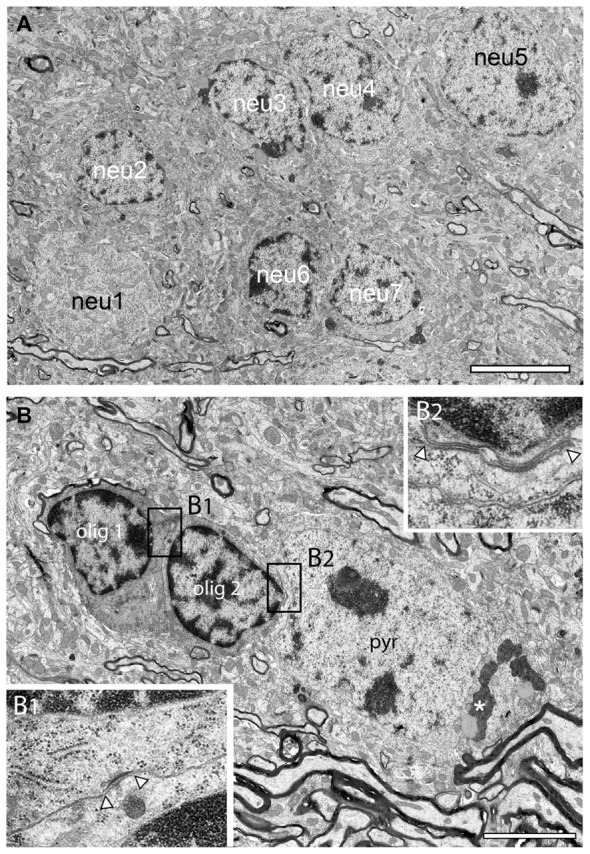
Neuronal clusters in the adult *reeler* mouse neocortical mass. **(A)** Large homologous cluster of seven, putative spiny stellate neurons (neu1–neu7) in the mid part of the neocortical mass as indicated by their comparably smaller size and distribution of the heterochromatin. **(B)** Typical example of a heterologous cluster between two oligodendrocytes (olig1, olig2) and a putative pyramidal neuron (pyr) located close to a massive axonal fiber bundle. The framed areas **(B1,B2)** show a tight-junction **(B1)** between the two oligodendrocytes and a gap-junction between olig 2 and the adjacent pyramidal (pyr) neuron **(B2)**. Note the appearance of lipofuscin granula (asterisk) in the cytoplasm of the pyramidal neuron but not in the oligodendrocytes. Scale bar 5 μm.

**Figure 8:**
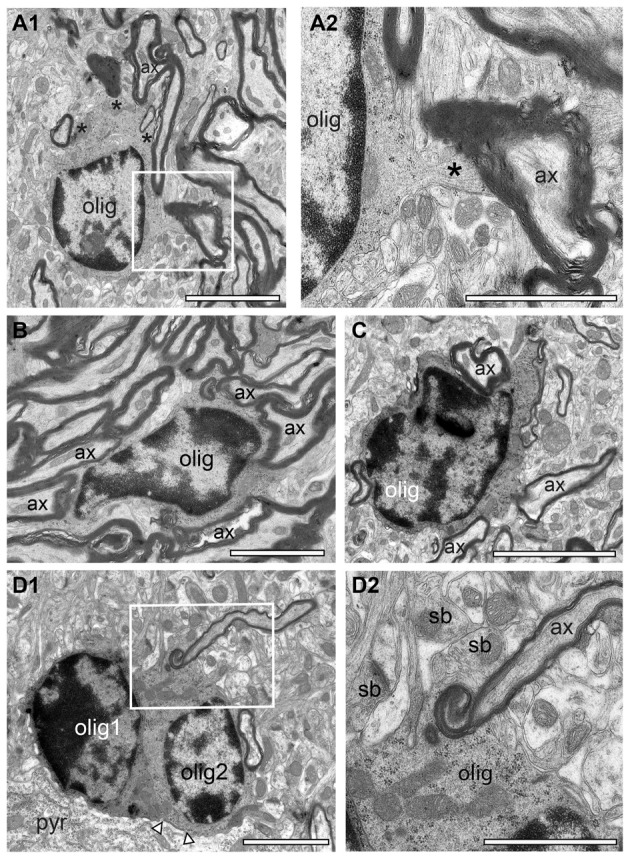
“Active” oligodendrocytes in the adult *reeler* mouse neocortical mass. **(A1)** Oligodendrocyte (olig) in the superficial part of the neocortex myelinating several axons (ax) as marked by asterisks. The framed area is shown at higher magnification in **(A2)**. Scale bar 10 μm. **(A2)** High power micrograph of a cytoplasmic finger (asterisk) of the oligodendrocyte (olig) shown in **(A1)** myelinating an axon (ax). Scale bar 20 μm. **(B,C)** Two oligodendrocytes located in the mid **(B)** and deep **(C)** portion of the neocortical mass. Both oligodendrocytes are seen to myelinate several axons. Scale bar in **(B)** 10 μm, **(C)** 20 μm. **(D1)** Pair of oligodendrocytes (olig1, olig2) next to the cytoplasm of a neuron (pyr) in the deep neocortical mass. Note that the right oligodendrocyte is coupled to the neuron via a gap-junction (arrowheads). The framed area is shown at higher magnification in **(D2)**. Scale bar 10 μm. **(D2)** High power micrograph of the framed area in **(D1)** showing the process of myelinating an axon (ax). Note several synaptic boutons (sb) in the adjacent neuropil. Scale bar 10 μm.

**Figure 9:**
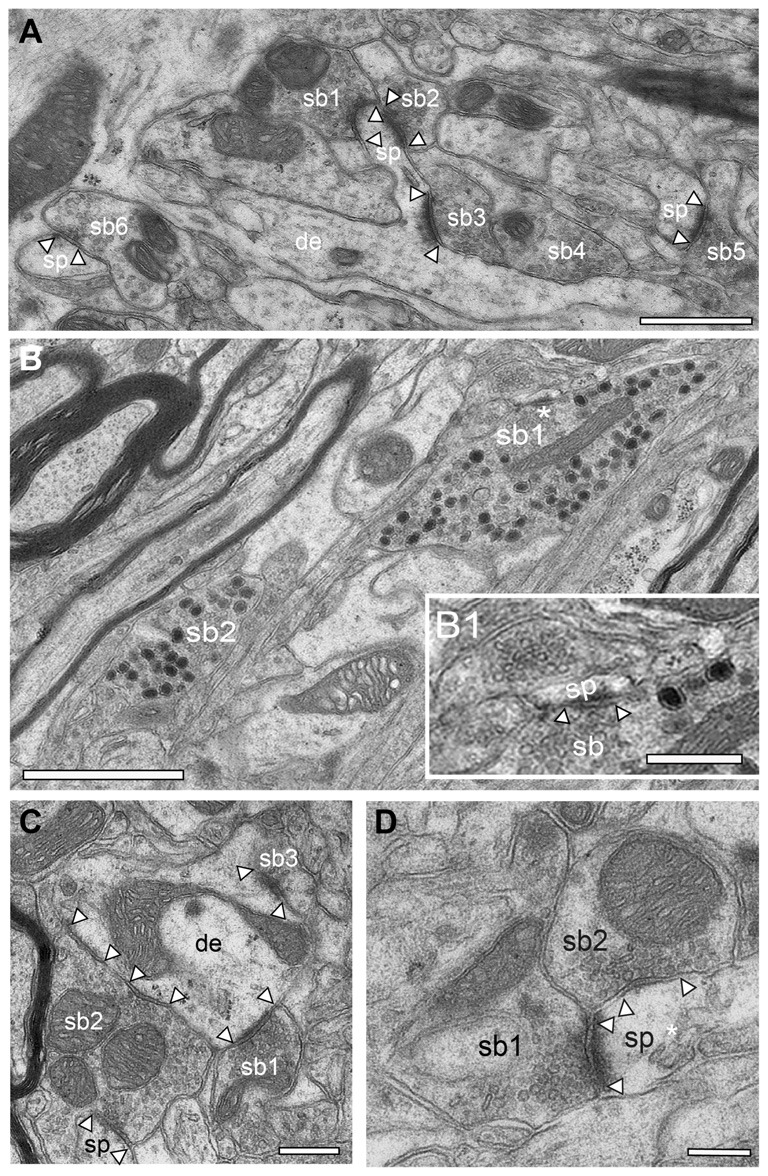
Synaptic organization of the adult *reeler* mouse neocortex. **(A)** Low power EM micrograph of a dendritic shaft (de) with an elongated spine (sp). Note the establishment of two synaptic boutons (sb1, sb2) on the spine head and one on a spine neck (sb3). In the surrounding neuropil several other synaptic boutons (sb4–6) terminating on spines (sp) were found. Scale bar 0.5 μm. **(B)** Two large synaptic boutons (sb1, sb2) located within a massive fiber bundle in the superficial part of the neocortical mass one of which is shown in the framed area in Figure 5B. Note the high number of large dense core vesicles distributed throughout the entire bouton. Scale bar 0.5 μm. **(B1)** High magnification of the region marked by an asterisk in **(B)** showing an active zone at a small spine (sp). Note also the two large dense core vesicles in close proximity to the active zone. Scale 0.25 μm. **(C)** Small caliber dendrite (de) with three synaptic boutons (sb1–3) two of which are putative glutamatergic terminals (sb1, sb3) as indicated by the shape of synaptic vesicles and that of the active zone. Sp2, a possibly GABAergic bouton innervates two postsynaptic targets, a dendrite (de) and a spine (sp). Scale bar 0.25 μm. **(D)** Double innervation of a spine (sp) by two different synaptic boutons (sb1 putative glutamatergic) and sb2 (putative GABAergic) as indicated by the shape and size of the active zone, the lack of a prominent PSD (sb2) and the shape of synaptic vesicles. The spine apparatus is marked by an asterisk. Scale bar 0.2 μm. In all images, active zones are marked by arrowheads.

In WT, L1 is ~5-fold larger in volume and clearly distinguishable as a distinct layer from the underlying L2/3 as indicated by the increasing density of neurons (Figure 6A). In addition, the neuropil of L1 is more densely packed but composed of the same structural subelements such as astrocytes (Figure 6B), dendrites (Figure 6B), and synaptic boutons (Figure 6C). When compared with* reeler*, GABAergic interneurons identifiable by the comparably smaller size when compared with pyramidal cells, their shape and nuclear enfolding were only occasionally found in L1 of WT (Figure 6B) and *reeler* mice. In both* reeler* and WT neurons were sometimes coupled via tight- (Figure 6D) or gap-junctions (Figure 6E).

### Neuronal Clusters in the Adult *Reeler* Mouse Neocortex

Instead of being organized into their prospective layers as in adult WT mice, neurons of a distinctive phenotype formed clusters throughout the entire neocortical mass in *reeler* (Figure 2B framed areas, Figure 7A). Homologous clusters of neurons (Figure 7A) were found in all parts of the neocortical mass and were comprised of several (3–10) neurons (Figure 2B framed areas, Figure 7A). In addition to homologous clusters, heterologous clusters composed of neurons and oligodendrocytes (Figure 7B) were also present throughout the* reeler* neocortical mass. Within both, homologous or heterologous clusters, neurons were often coupled via tight- (Figure 7B1) or gap-junctions with oligodendrocytes (Figures 7B2, 8D1). Upon established ultrastructural criteria (Peters et al., 1991) neurons building clusters were excitatory in nature according to the shape and size of their nuclei, the distribution of heterochromatin within nuclei, the presence of a nucleolus, and the organization of their cytoplasm and their size when compared with GABAergic interneurons (compare Figure 5B with Figure 6B). No such organization of neurons was found in adult WT mice where neurons are located in distinct layers (Figure 6A). One common structural feature of neurons in adult WT and *reeler* was the appearence of lysosomes, lipid bodies and lipofuscin granula within the cytoplasm as a sign of aging (Figures 4C, 7B).

### “Active” Oligodendrocytes in the Adult *Reeler* Mouse and WT

Oligodendrocytes are the source of myelin sheaths in the CNS allowing rapid saltatory conduction and also providing metabolic support to neurons. It is well established that in the adult WT CNS oligodendrocytes were exclusively found in fiber tracts, for example in the white matter. In contrast, in adult *reeler*, oligodendrocytes were located throughout the entire neocortical mass (Figures 3C,D, 5B, 7B), and the white matter (Figure 8). They were seen as single or groups of 3–7 oligodendrocytes within fascicles of fibers (Figures 3C,D, 8) or at the edges of myelinated fiber bundles (Figure 8A1). Oligodendrocytes closely attached to each other were often coupled via gap-junctions (Figure 7B2).

Interestingly, nearly all oligodendrocytes investigated were seen to produce myelin as indicated by several myelinated axons either attached (Figure 8A2) or found within the cytoplasm (Figures 8D1,D2) also indicative for ongoing myelination regardless of their location in the neocortical mass in *reeler*. No such “active” oligodendrocytes were found in the gray matter of WT mice. Here, only a few oligodendrocytes were seen that are closely attached to myelinated fibers (compare Figures 4A,D with Figure 8).

### Synaptic Organization in the Adult *Reeler* Mouse Neocortex

**Figure 10:**
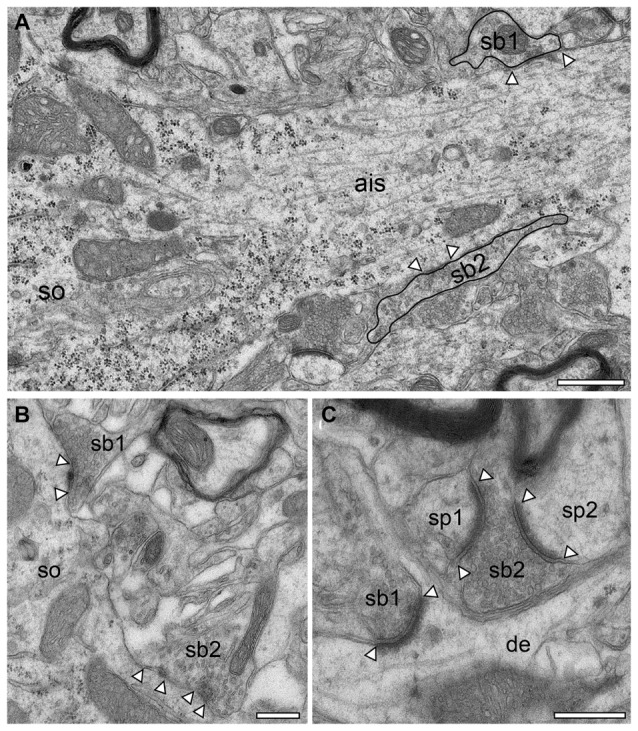
Synapses in the adult* reeler* mouse neocortex. **(A)** Axon initial segment (ais) emerging directly from the soma (so) of a putative pyramidal neuron with two terminating synaptic boutons (sb1, sb2, highlighted by black contours). Scale bar 0.5 μm. **(B)** Two somatic (so) putative GABAergic synaptic boutons (sb1, sb2) located in the mid part of the neocortical mass, one with two active zones. **(C)** Two putative glutamatergic synaptic boutons (sb1, sb2) terminating on different targets. Sb1 is located at the emergence zone of a filopodial spine close to the dendrite (de). Sb2 is terminating on two opposing spines (sp1, sp2). Scale bar in **(B,C)** 2 μm. In all images, active zones are marked by arrowheads.

**Figure 11:**
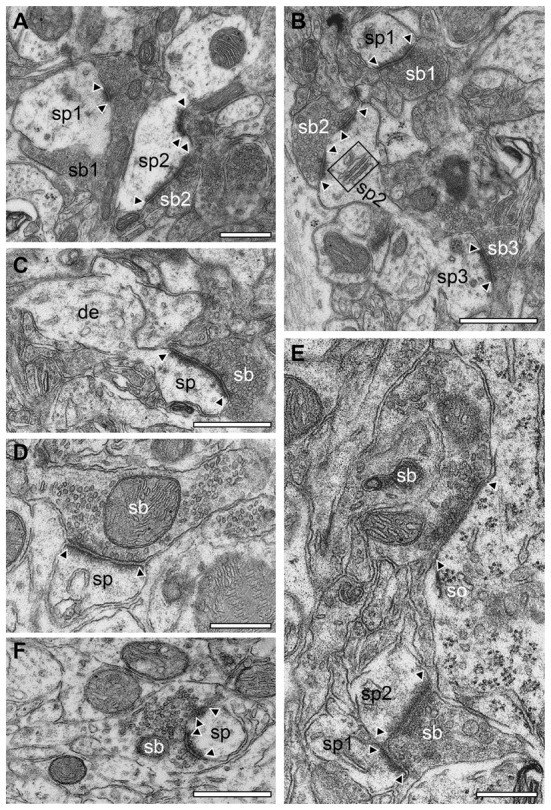
Synapses in different layers of the adult WT mouse neocortex. **(A)** EM micrograph of two synaptic complexes between two spines (sp1, sp2) of different shape and size and two synaptic boutons (sb1, sb2) in L2/3. Note that sb1 was comparably large in both size and its pool of synaptic vesicles but had only a single small active zone whereas sb2 had two active zones located at the spine head and neck and a smaller pool of synaptic vesicles. **(B)** Two opposing spines (sp2, sp3) located on the same dendrite with two synaptic boutons (sb2, sb3) in L2/3. Sp3 had only a single active zone whereas sp2 contained two active zones and a prominent spine apparatus (framed area). In the surrounding neuropil another synaptic complex (sp1, sb1) with a single, but large active zone covering almost the entire length of the synaptic apposition zone is shown. **(C)** Synaptic bouton (sb) terminating on a typical mushroom-shaped spine (sp) emerging from a small caliber dendrite (de) with a large non-perforated active zone in L4. **(D)** *En passent* putative GABAergic synaptic bouton (sb) identified by the small ovoid-shaped synaptic vesicles and the narrow PSD of the active zone terminating on a spine (sp) in L4. **(E)** GABAergic synaptic bouton (sb) terminating on the soma (so) of a putative pyramidal neuron in L5. Note also the synaptic bouton (sb) terminating on two adjacent spines (sp1, sp2). **(F)** Synaptic bouton (sb) with two active zones completely ensheathing a dendritic spine head (sp) located in L6. Scale bar in **(A–F)** 0.5 μm. In all images, active zones are marked by arrowheads.

Synapses are key elements in the induction, maintenance and termination of synaptic transmission and in modulating synaptic plasticity (Südhof, 2012). Is the absence of layers, the malpositioning (clustering) of neurons and their different polarity also accompanied by differences in the composition and alterations in the postsynaptic innervation pattern of neurons by synaptic boutons? To address this question the structure and postsynaptic targets of synaptic boutons throughout the adult *reeler* somatosensory neocortex were qualitatively investigated (Figures 9, 10) and compared with the WT (Figure 11).

### General Observations

In general, in the adult *reeler* mouse neocortex synaptic boutons displayed no obvious alterations and distortions in their structural composition and specific postsynaptic innervation pattern on their respective target structures (Figures 9, 10). Synaptic boutons of different shape and size were found throughout the neuropil of the superficial (Figure 5B inset, Figures 9A,B), mid (Figures 9C,D) and deep gray matter of the neocortical mass (Figures 10B,C). Both types, endterminal (Figures 9A,C,D, 10B,C) and *en passant* (Figure 10A) synaptic boutons were frequently observed. On average they contained, a single, often very large, mostly non-perforated active zone (Figures 9A,D, 10C), but occasionally perforations of either the pre- or postsynaptic density or both were seen (Figure 10B). In a few synaptic boutons more than one, at most three, active zones were found (Figure 9C).

In addition, synaptic boutons were highly variable in their content of synaptic vesicles that were distributed throughout the entire terminal and often densely packed (Figures 9C, 10C), in particular at the active zones. Beside those with only a comparably small pool of synaptic vesicles (Figures 9A,D) comparably large total pools of synaptic vesicles (Figures 9B,C, 10C) were found by qualitative inspection. In the majority of synaptic boutons several so-called “docked vesicles” (2–7) attached or fused with the presynaptic density could be demonstrated (Figures 9B,D) pointing to multivesicular release at these synapses. Single or several mitochondria of different shape and size were frequently observed in the presynaptic bouton in close association with the synaptic vesicles pool (Figures 9C,D, 10B).

The majority of synaptic contacts were established on spines of different types including filopodial (Figure 9A), mushroom (Figures 9D, 10C), and stubby (not shown) spines. They often contained, as described for WT (Figure 6C framed area, Figure 11B framed area), a spine apparatus, a highly specialized derivate of the endoplasmic reticulum (Figure 5B inset, Figures 9A,D in *reeler*). Spines containing a spine apparatus are more mobile and are thought to partially contribute in the modulation of short- and long-term plasticity (Konur and Yuste, 2004a,b; Holtmaat et al., 2005).

Strikingly, exceptionally large synaptic boutons containing numerous dense-core vesicles were present exclusively in the most superficial part of the neocortical mass in *reeler* (Figure 9B). There, they were found mainly between the myelinated axonal bundles (Figure 9B). These terminals established several synaptic contacts mainly with dendritic spines (Figure 9B). Most of the dense-core vesicles were distributed throughout the entire terminal (Figure 9B). However, some were located in close proximity to the active zone (Figure 9B1). Hence it has been suggested that besides being involved in exo- and endocytosis (Watanabe et al., 2013), a subpopulation of dense-core vesicles contains and releases *Piccolo and Basson*, two proteins involved in the build-up of the presynaptic density (Shapira et al., 2003; Gundelfinger et al., 2015), or by clustering SVs at the PreAZs (Mukherjee et al., 2010; Watanabe et al., 2013). In addition various co-transmitters, such as neuropeptides, ATP, noradrenalin, and dynorphin can be packed in large dense-core vesicles (Ghijsen and Leenders, 2005; Zhang et al., 2011).

Synaptic boutons containing dense-core vesicles were also found in WT mice throughout all cortical layers, but seem to contain a much lower number of these vesicles.

No obvious differences in the shape and size of synaptic complexes, and postsynaptic innervation pattern were found in WT (Figure 11) compared to *reeler* mice (Figures 9, 10). In general, endterminal (Figures 11A–C,E,F) and *en passant* (Figure 11D) synaptic boutons established synaptic contacts on dendritic shafts, spines of different caliber and shape (Figure 11) and somata (Figure 11E). Interestingly, also a few GABAergic terminals were found on dendritic spines in both *reeler* and WT mice (compare Figure 9D with Figure 11D).

Throughout the neuropil, a dense network of astrocytes and their processes was present in order to isolate adjacent synaptic complexes from each other (Figures 9A–D, 10B,C). No obvious differences in the astrocytic coverage of synaptic complexes were found between *reeler* and WT mice (Prume et al., paper in preparation).

### Postsynaptic Targets of Synaptic Boutons in Adult *Reeler* and WT Mice

Dendrites of different caliber usually received dense synaptic input directly on their shafts (Figures 9A,C) or spines (Figures 9A,B,D, 10C) in both *reeler* and WT mice. Occasionally, synaptic boutons were seen to establish a synaptic contact at the spine neck (Figures 9A, 10C) or head (Figures 9A,D). Sometimes spines were innervated by two (Figure 9A), at most three synaptic boutons, mainly located on the spine head. Interestingly, a side-by-side termination of glutamatergic and GABAergic synaptic boutons was observed on both dendritic shafts (Figure 9C) and rarely even on spines (Figure 9D; see also Kwon et al., 2018). In *reeler* and WT mice GABAergic terminals were identified by the lack of a prominent postsynaptic density and the smaller more oval shaped synaptic vesicles (Figures 9C,D, 10B, 11D). Some of the GABAergic terminals were extremely large and elongated and contained a large pool of synaptic vesicles (Figure 10A, sb2). In addition, numerous synaptic boutons of the *en passant* (Figure 10A) and endterminal type terminated on somata of neurons throughout the entire neocortex (Figure 10B) as well as at the axon initial segment (Figure 10A).

In summary, the synaptic organization in the adult *reeler* mouse neocortex exhibited the same structural composition of synaptic boutons and postsynaptic innervation pattern as described and shown in adult WT mice (Figure 11).

## Discussion

This study shows in detail the neuronal and synaptic organization of the adult *reeler* mouse somatosensory neocortex compared to that of the WT using high-resolution, fine-scale EM. Despite the lack of a six-layered structure composed of distinct populations of neurons characteristic for the neocortex in WT mice, the neocortical mass in *reeler* is organized in homologous clusters (islands) of neurons with maintained morphological identity and heterologous clusters of neurons and oligodendrocytes. When compared with WT mice, the *reeler* mutation displays both a “normal” structural composition of synaptic boutons with no obvious sign of degeneration and distortions and a “normal” postsynaptic innervation pattern of neurons by these boutons (compare Figures 9, 10 with Figure 11). This provides the structural basis for the establishment of a functional although altered cortical network in adult *reeler* (Wagener et al., 2010, 2016; Guy et al., 2015, 2017; Pielecka-Fortuna et al., 2015).

### Does L1 Exist in the Adult *Reeler* Mouse Neocortex?

It is still controversially discussed whether a substantial L1 exists in *reeler* or whether this layer is diluted into the volume of the underlying neocortical mass. In adult WT species including rodents, L1 is regarded as a “fiber” and cell-sparse layer containing a dense network of astrocytes, different types of GABAergic interneurons (Zuchero and Barres, 2015; Lee and D’Arcangelo, 2016; Yao et al., 2016), commissural and associational axons and terminal tuft dendrites of pyramidal neurons (Marín-Padilla, 1978, 1998) and thus distinguishable from L2/3 by the dramatically increasing density of neurons (Figure 6A).

In adult *reeler*, however, some previous experimental findings support the existence of an, although “altered” L1. First, during early cortical development L1, a derivate of the marginal zone is one of the earliest generated layers in WT mice, whereas in *reeler* L1, if existent, is constituted of the superplate composed of the marginal zone and subplate (reviewed by Bystron et al., 2008). Second, Cajal-Retzius cells, a transient population of early born neurons exclusively located in L1 in WT mice (Anstötz et al., 2014) are also exclusively found in the most superficial part in *reeler* closely attached to the pial surface but with a comparably lower density (Anstötz, unpublished observation). As in WT, the occurrence of Cajal-Retzius cells in *reeler* points to the existence of L1.

Finally, L1 in adult WT mice contains subpopulations of GABAergic interneurons, a subset of these neurons is labeled by A930038C07Rik and the cholinergic receptor Chrna7 (Boyle et al., 2011). In *reeler*, neurons expressing the two genes are localized at the pial surface/most superficial part of the neocortical mass with a strong expression from E18.5 through adulthood. However, they are also located throughout the upper and mid cortical region (cortical plate) in primary sensory areas from P7 onwards in *reeler*. This indicates either a postnatal migration of these neurons from the pial surface into the neocortical mass or a new ectopic population of neurons expressing these genes (Boyle et al., 2011).

In adult *reeler*, L1 if existent, was severely reduced causally related to the loss of a *Reelin*-mediated stop signal via VLDL-receptors. In addition, *Reelin* is also involved in the migration stop by Dab1 degradation via the SOCS7-Cullin5-Rbx2 system. Finally, *Reelin* also controls the terminal translocation of neurons from radial glial fibers which in* reeler* is severely obstructed (Pinto-Lord et al., 1982) via activation of cell adhesion molecules (Sekine et al., 2014).

Altogether, the lack of *Reelin*-mediated signaling cascades may explain the massive invasion by excitatory, putative pyramidal neurons from the underlying neocortical mass into the most superficial part. These neurons establish synaptic contacts with GABAergic interneurons, themselves and other neurons in the underlying neocortical mass thereby contributing to “additional” new networks (microcircuits) in the neocortical mass in *reeler* (Radnikow et al., unpublished observations). However, the existence and functional properties of such networks has yet to be investigated using for example paired recordings from synaptically coupled neurons or optogenetic approaches in *reeler*.

### Fiber Bundles in the Adult *Reeler* Mouse Neocortex

Another striking feature of the most superficial part of the cortical mass in *reeler* are massive, long-range horizontally oriented myelinated axons, running parallel to the pial surface and then descending throughout the entire neocortical mass. These fiber bundles are prominent in osmium-treated sections (Figure 1 this study) but never found throughout cortical L1 to L6A in WT where fibers seemed to be more randomly distributed with a much lower density. It has to be noted that such massive fiber bundles organized in fascicles were exclusively found in L6B emerging from the white matter in WT mice.

Such massive fiber bundles in *reeler* were first demonstrated light microscopically by Caviness and Yorke (1976) and later confirmed by Goffinet (1980) as axonal trajectories from the white matter to the superficial part of the neocortical mass. In addition, Caviness et al. (1972) hypothesized that these “thalamic” axons seemed to approach their target neurons from above (superficial) towards L1. This hypothesis was later confirmed by Molnár et al. and co-workers (Molnár et al., 1998, 2003; López-Bendito and Molnár, 2003; Higashi et al., 2005) by tracing the thalamocortical fiber projections.

This study supports and further extends this view at the fine-scale EM level demonstrating that ascending and descending thalamocortical fiber bundles were found throughout the gray matter of the neocortical mass. As a novel, yet not described finding these fiber bundles were always accompanied by clusters of “active” oligodendrocytes (see Figures 3–6), still in the process of myelination. In contrast, we can clearly demonstrate that oligodendrocytes were not observed in the gray matter of WT mice. In addition, oligodendrocytes in the white matter of WT mice were only rarely seen with myelinated fibers attached to their cytoplasm indicative for ongoing myelination (compare Figures 8D1,D2 with Figure 4D) and were thus regarded as “silent.” It might be speculated, that such high numbers of “active” oligodendrocytes and massive unmyelinated and myelinated axons are required for the maintenance of the thalamocortical and intracortical axonal fiber bundles in *reeler*. This could lead to an imbalance of intracortical vs. thalamocortical inputs and may thus contribute to either a possible impairment or more likely to facilitation of intracortical network activity resulting in epileptic seizures observed in *reeler* (Patrylo et al., 2006).

### Structural Composition of the Somatosensory Neocortex in *Reeler*

Earlier studies have mainly focused on the cytoarchitectonic and synaptic organization of the developing *reeler* mouse neocortex including a few EM studies (Caviness et al., 1972; Welt and Steindler, 1977; Caviness and Rakic, 1978; Derer, 1979; Goffinet, 1980; Caviness, 1982; Pinto-Lord et al., 1982).

Here, we demonstrate that neurons of the “same” morphological identity (characterized by morphological criteria at the subcellular fine-scale EM level introduced by Peters et al., 1991) form homologous or heterologous clusters or islands. These findings are in line and thus support the protomap theory, a primordial molecular map of the functional areas of the mammalian cerebral cortex during early embryonic development in that the genetic fate of neurons is independent from the formation of layers (Rakic, 1988; reviewed by Rakic, 2009). Moreover, neurons involved in any given network of the brain should receive synaptic input and in turn provide synaptic output, a requirement for circuit formation, which seems to be “unaltered” in the adult *reeler* mouse neocortex. We can demonstrate that synaptic boutons show an “identical” structural composition with the same variability when compared with WT mice. Moreover, the postsynaptic innervation pattern also remains “unaltered” compared to that described in WT mice.

Interestingly, we often found neuronal clusters next to thalamocortical axons suggesting that these clusters may represent RORb-expressing cells receiving synaptic input from the thalamus (Jabaudon et al., 2012; Guy et al., 2015). RORb is an orphan-like receptor expressed by L4 neurons during development causally related to the formation of the “barrel field” and guiding thalamocortical afferents to their appropriate target neurons. However, it is still unclear whether RORb is expressed also in other than L4 neurons, and whether clustering of neurons are only observed by RORb overexpression *in vivo* (Jabaudon et al., 2012), although it does not explain the formation of heterologous clusters as shown in this study.

We might speculate that these neuronal clusters or “islands” of neurons may represent a possible “alternative” organization principle in adult *reeler* “replacing” layer formation. If so, these homologous clusters may thus represent individual “functional units” where neurons are highly interconnected and may function as the equivalent of neurons integrated in a respective layer of the cortical column. Evidence for such a scenario is that neurons in such clusters are highly interconnected via tight- or gap junctions, which may represent also electrical coupling between neurons. However, this hypothesis remains to be investigated for example by paired recordings of such neuronal clusters combined with intracellular biocytin-fillings in future correlated structural and functional studies.

## Conclusion and Future Prospects

Which structural elements are required to build a neuronal network? Synapses are key elements in any given network of the brain. In this study, we demonstrate that the innervation of postsynaptic target structures remains “normal” and “specific” in* reeler* as in the WT neocortex.

However, it is still unclear whether the lack of *Reelin*, has consequences on the density, distribution pattern and quantitative geometry of synaptic complexes in reeler, in particular the size and shape of active zones (transmitter release sites) and the size and organization of synaptic vesicles in the three functionally defined pools of synaptic vesicles, namely the readily releasable, the recycling and reserve pool. This will be addressed in a quantitative follow-up study (Prume et al., in preparation). Quantitative 3D-models of synaptic boutons and their target structures analyzing structural parameters that represent morphological correlates for synaptic transmission and plasticity will provide a further basis helping to understand synaptic “behavior” in the adult *reeler* mouse somatosensory neocortex when compared with WT mice.

Second, the axonal projection patterns and possible synaptic connectivity of neurons underlying the establishment of individual microcircuits building networks in the *reeler* mouse neocortex are largely unknown (but see for example Pielecka-Fortuna et al., 2015; Wagener et al., 2016). Thus, it would be of great importance to unravel such individual microcircuits using for example paired recordings or optogenetic approaches to identify individual synaptic connections in combination with intracellular biocytin-fillings. As one line of evidence for a “correct” wiring in *reeler*, thalamocortical afferents drive L4 excitatory spiny neurons by establishing functional synapses as in the WT neocortex (Wagener et al., 2016). However, whether such a correct wiring or synaptic connectivity as described by numerous publications in WT rodent neocortex (reviewed by Lübke and Feldmeyer, 2007; Feldmeyer et al., 2013) is preserved in the *reeler* neocortical mass remains largely unknown and has thus to be structurally and functionally investigated in future studies for example by patch-clamp recordings of synaptically coupled neurons or optogenetic approaches.

## Author Contributions

MP, AR and JL performed all structural experiments, electron microscopic data acquisition and analysis. JL and AR are responsible for the conception and writing of the article. The study was supported by the Helmholz Society (budget to JL).

## Conflict of Interest Statement

The authors declare that the research was conducted in the absence of any commercial or financial relationships that could be construed as a potential conflict of interest.
